# The effect of breathing exercises on adults' sleep quality: an intervention that works

**DOI:** 10.3389/frsle.2025.1603713

**Published:** 2025-06-25

**Authors:** Valērija Steinmane, Andra Fernate

**Affiliations:** Latvian Academy of Sport Education, Riga Stradins University, Riga, Latvia

**Keywords:** breathing exercises, effect of breathing exercises, mindful breathing, sleep regulation, sleep quality

## Abstract

This study investigates the application of various breathing exercises to enhance sleep quality. Respiration is an indispensable aspect of life that significantly influences both the physical and mental wellbeing of individuals, as well as sleep quality. Embracing appropriate breathing exercises has proven to be advantageous for short-term relief and long-term holistic health. The aim of the study is to theoretically investigate the application of breathing exercises to improve sleep quality. Scientific article review was conducted, drawing upon completed trials within scientific articles found in ScienceDirect, PubMed and Scopus databases (period 2000–2024). The focus on articles that utilize deep breathing, diaphragmatic breathing, mindful breathing, respiratory muscle training, and pursed lip breathing allowed us to explore the potential benefits of these techniques on sleep quality. By synthesizing findings from diverse trials, this study contributes to a deeper understanding of how specific breathing techniques may modulate physiological and psychological factors involved in sleep regulation. The durations of adult engagement in daily breathing exercise sessions lasted for a month or longer. Study results show that consistent success was observed across seven studies employing various breathing exercises for distinct patient groups, with improved sleep quality noted among participants who completed the prescribed breathing techniques. The insights gained from this theoretical investigation promote knowledge for an intervention (content, duration, timing) that works and improves sleep quality through the approach of breathing exercise application.

## 1 Introduction

A growing number of studies have proved that breathing exercises can significantly improve the quality of life (Tsai et al., 1995). Study suggests that a breathing exercise can be safely used and may have positive effects on the posture, flexibility and strength of healthy adults (Csepregi et al., 2022).

It is known that breathing exercises covers a multitude of therapeutic approaches, however, the meaning of the breathing exercises is not generally defined (Bruton et al., 2011). There are many different views, but there is a lack of definitions for known breathing exercises. Trials involving breathing techniques have historically been published simply describe the intervention as involving a course of breathing exercises. Although most authors now provide more detail, it is still generally insufficient to allow accurate replication.

The aim of this research is to conduct an interdisciplinary (medicine, physiology) literature review on the meaning of breathing exercises and its classification. The aim will be reached by researching scientific articles on PubMed, ScienceDirect and Scopus databases for the concept of breathing exercises as well as to define terminology and analyze the scientific literature and previous scientific studies that can define and classify the meaning of breathing exercises.

“Breathing exercises” is a phrase which is used by scientists and therapists to describe aim and functions, but not the meaning of the exercises. Interventions used in clinical practice and research need to be described in sufficient detail to permit accurate replication.

Current study is connected to previously conducted studies by the approach on breathing exercise application for improvement of quality of life. Previous studies produced eight breathing exercise components that serve as the basis for the current study.

Several conducted studies show that breathing exercises can show different results (Laís et al., 2019; Hamasaki, 2020; Fink, 2007; Betka et al., 2022; Cecins et al., 1999) depending on the approach to exercise application. Since words and phrases can change their meaning over time, it is important that authors choose their words carefully and define anything which might be ambiguous. Unfortunately, no clear classification of breathing exercises is available.

## 2 Materials and methods

A systematic search was conducted using PubMed, ScienceDirect, and Scopus databases to identify relevant studies published between 2000 and 2024. The search strategy included the keywords:

- “breathing exercises” and “sleep quality”
- “respiratory training” and “sleep improvement”
- “deep breathing” and “insomnia treatment”

**Inclusion criteria:**

- Peer-reviewed articles published in English
- Systematic reviews, clinical trials, observational studies and research articles
- Studies including adult participants (aged 18+)
- Research explicitly evaluating the intervention impact of breathing exercises on sleep quality

**Exclusion criteria:**

- Studies focusing on children or adolescents or animals
- Research on sleep disorders unrelated to breathing techniques
- Studies with insufficient methodology or lacking control groups
- Studies lacking full-text access
- Studies not centered on breathing as the main intervention

The review identified key studies focusing on various breathing exercises, their duration, frequency, and observed effects.

## 3 Results

**Figure 1:**
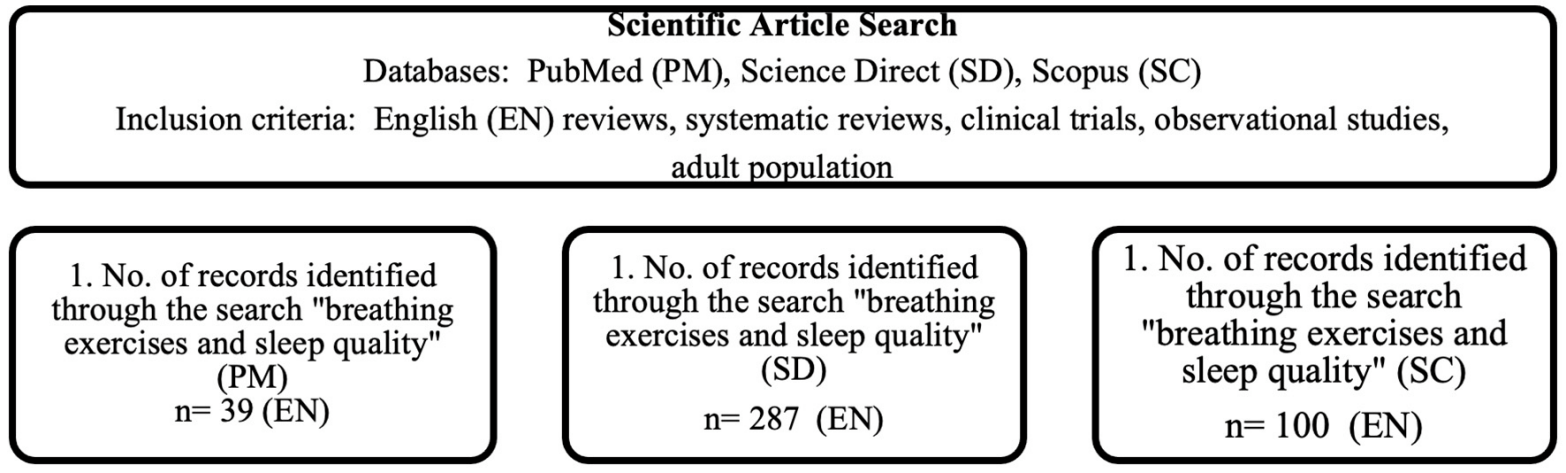
The process of systematic search (by the authors).

The results of the research provided a list of breathing exercises that was found to have positive impact on sleep quality. Research produced description of breathing exercise, its duration, repetition amount and exercise duration, as well as experiment group that allow to better understand the effect of the breathing exercises. This approach allowed to gather comprehensive data on the application of breathing exercises for improving sleep quality within the specified timeframe and target demographic. As shown in Figure 1, the search for keywords resulted in broad range of articles that mention breathing exercises and sleep quality; however, only a limited number of articles corresponds to the purpose of the study.

Search within PubMed database produced 39 articles for requested keywords, search within ScienceDirect database produced 287 articles and search within Scopus database produced 100 articles. The systematic review identified six key studies that demonstrate the positive impact of breathing exercises on sleep quality. These studies included clinical trials, randomized controlled trials, and systematic reviews analyzing the effects of diaphragmatic breathing, mindful breathing, deep breathing, and respiratory muscle training (Figure 1).

The search within the PubMed (PM), Science Direct (SD) and Scopus (SC) databases for the keywords “Breathing exercises and sleep quality” with set limitations (publishing years 2000–2024; study type: review, systematic review, full-text; language: English) produced the following breathing exercise use case (Table 1, Line 1): This randomized controlled study was conducted with three groups: pranayama, deep breathing, and control group, each group contained 20 patients. 10-min sessions of deep breathing exercises were conducted after radiation sessions for five consecutive weeks, altogether patients made 25 sessions. Sleep quality was improved. The Pittsburgh Sleep Quality Index were used to evaluate the participants' fatigue and sleep quality.

**Table 1:** Summary of breathing exercise studies and their impact on sleep quality (by the authors).

**Research authors**	**Exercise type**	**Duration**	**Repetitions**	**Duration of the exercise session**	**Result**	**Patient diagnose**
Gündogdu and Koçaşli (2023)	Deep breathing	5 weeks	25 sessions	10 min	Sleep quality was improved	Woman with breast cancer
Anderson and Bliven (2017)	Diaphragmatic breathing	4 weeks	12 sessions	-	Sleep quality was improved	Chronic low back pain
Hui et al. (2021)	Mindful breathing	1–2 months	Daily	30 min	Significant improvements in sleep quality	Insomnia
Kuo et al. (2017)	Respiratory muscle training	5 weeks	-	-	Improved sleep apnea	Obstructive sleep apnea
Liu et al. (2021)	Diaphragmatic breathing	4 weeks	Daily	20 min	Is effective for improving sleep quality and reducing anxiety	Nursing staff during COVID-19 outbreak
Fadi et al. (2020)	Pursed lip breathing, Diaphragmatic breathing	4 weeks	Daily	1 h	Had significance effect on improving sleeping quality	Hospitalized patients, random diagnosis

The search within the PubMed (PM), Science Direct (SD) and Scopus (SC) databases for the keywords “Breathing exercises and sleep quality” with set limitations (publishing years 2000–2024; study type: review, systematic review, full-text; language: English) produced the following breathing exercise use case (Table 1, Line 2): The purpose of this study was to test the effect of adding diaphragmatic breathing exercises to core stabilization exercises for patients with chronic low back pain. 22 patients were randomly allocated. They were given 12 treatment sessions 3 times a week for 4 weeks. Sleep quality was improved. The Pittsburgh Sleep Quality Index were used to evaluate the participants' sleep quality.

The search within the PubMed (PM), Science Direct (SD) and Scopus (SC) databases for the keywords “Breathing exercises and sleep quality” with set limitations (publishing years 2000–2024; study type: review, systematic review, full-text; language: English) produced the following breathing exercise use case (Table 1, Line 3): 40 patients were assigned to the control group, and 40 patients to the treatment group. The control group received routine pharmacological and physical therapies, while the treatment group received instructions to add mindful breathing exercises to the routine therapies. The results showed that mindful breathing combined with the sleep-inducing exercise significantly improved the long-term effectiveness of insomnia treatment.

The search within the PubMed (PM), Science Direct (SD) and Scopus (SC) databases for the keywords “Breathing exercises and sleep quality” with set limitations (publishing years 2000–2024; study type: review, systematic review, full-text; language: English) produced the following breathing exercise use case (Table 1, Line 4): The study investigated the effects of 5 weeks of expiratory muscle strength training on sleep apnea and sleep quality. This investigation demonstrated that 5 weeks of respiratory muscle training using an expiratory muscle strength training improved sleep apnea.

The search within the PubMed (PM), Science Direct (SD) and Scopus (SC) databases for the keywords “Breathing exercises and sleep quality” with set limitations (publishing years 2000–2024; study type: review, systematic review, full-text; language: English) produced the following breathing exercise use case (Table 1, Line 5): The definition found within the PM database gives a clear description of the breathing exercise in question, as well as describes the effect of the exercise. However, no information is provided on what are the advantages of using the exercises included in the definition. In a similar manner, the SD database gives a brief description of the technique, but it does not provide any details of the effect of the exercises.

The search within the PubMed (PM), Science Direct (SD) and Scopus (SC) databases for the keywords “Breathing exercises and sleep quality” with set limitations (publishing years 2000–2024; study type: review, systematic review, full-text; language: English) produced the following breathing exercise use case (Table 1, Line 6): The study aimed to evaluate the effectiveness of diaphragmatic breathing relaxation training for improving sleep quality among nurses. 151 first line nurses participated in the research for 4 weeks conducting diaphragmatic breathing exercises daily for 20 min. Breathing exercises were proved to be effective for improving sleep quality and reducing anxiety.

## 4 Discussion

This study highlights the significant role of breathing exercises in improving sleep quality across various populations (Gündogdu and Koçaşli, 2023; Liu et al., 2021; Anderson and Bliven, 2017). These techniques work through key mechanisms including autonomic nervous system modulation, stress reduction, and enhanced respiratory function. Controlled breathing activates the parasympathetic nervous system while reducing sympathetic activity, fostering a physiological state conducive to sleep. This autonomic shift leads to reduced heart rate and blood pressure, supporting sleep onset and maintenance (Hui et al., 2021; Kuo et al., 2017). Moreover, breathing exercises help regulate the hypothalamic-pituitary-adrenal (HPA) axis by lowering cortisol levels, reducing stress and anxiety common contributors to insomnia and sleep fragmentation (Hui et al., 2021; Liu et al., 2021).

Improvements in respiratory efficiency also make breathing techniques beneficial for individuals with sleep-disordered breathing, such as obstructive sleep apnea (Kuo et al., 2017; Anderson and Bliven, 2017). Strengthened respiratory muscles and better oxygenation help reduce apneas and promote deeper, more restorative sleep.

Given their low cost, accessibility, and minimal risk, breathing exercises represent a valuable, non-pharmacological tool for enhancing sleep (Hui et al., 2021; Liu et al., 2021). Future research should aim to standardize breathing exercise protocols, identifying the most effective techniques, durations, and frequencies for different populations and sleep disorders. Longitudinal studies are needed to assess the long-term benefits and adherence to breathing interventions. Additionally, integrating breathing exercises with other non-pharmacological approaches such as cognitive-behavioral therapy for insomnia (CBT-I) or mindfulness-based stress reduction may enhance therapeutic outcomes. Exploring neurophysiological markers and individual variability in response to breathing practices could also help tailor personalized sleep improvement strategies.

## 5 Challenges and limitations

While the current research provides compelling evidence for the benefits of breathing exercises, several limitations need to be addressed before these techniques can be fully integrated into mainstream sleep interventions:

- Variability in study designs: studies on breathing exercises and sleep vary significantly in terms of methodology, participant demographics, and the specific techniques used. This lack of consistency makes it difficult to compare results across different studies and draw definitive conclusions.
- Limited long-term research: many studies focus on the short-term effects of breathing exercises, with limited data on their long-term sustainability. More research is needed to determine whether these benefits persist over extended periods.
- Lack of standardized protocols: there is currently no universally accepted guideline for how often, how long, and which specific breathing techniques should be practiced to achieve optimal sleep benefits. Establishing standardized, evidence-based protocols would help make breathing exercises a more reliable sleep intervention.

## 6 Future directions

To fully harness the potential of breathing exercises for sleep improvement, future research should focus on:

- Conducting large-scale, long-term randomized controlled trials to better understand the lasting impact of breathing exercises on sleep.
- Investigating the influence of demographic and psychological factors, such as age, gender, and pre-existing mental health conditions, in shaping the effectiveness of these interventions.
- Developing standardized, evidence-based breathing exercise protocols that can be easily adopted in both clinical settings and at-home practices.

## 7 Conclusion

Breathing exercises represent a promising, non-pharmacological intervention for improving sleep quality, offering a natural and accessible alternative to pharmaceutical sleep aids. The current evidence highlights the significant potential of controlled breathing techniques to enhance sleep by promoting relaxation, reducing stress, and improving respiratory function. These exercises target key physiological mechanisms, such as the regulation of the autonomic nervous system, which helps facilitate the transition into restful sleep by stimulating the parasympathetic response and mitigating the effects of stress.

The findings from the reviewed studies emphasize that breathing exercises can play a crucial role in improving sleep quality across various adult populations. From diaphragmatic and deep breathing to respiratory muscle training and mindful breathing, each technique has demonstrated the potential to positively influence sleep outcomes. However, despite the promising results, there remains a need for more rigorous research to refine these techniques and establish best practices. Variability in study designs, limited long-term research, and the lack of standardized protocols hinder the ability to draw definitive conclusions regarding the most effective breathing exercises for sleep quality.

To align with the thesis of this article, the evidence gathered clearly supports the notion that breathing exercises can be an effective, non-invasive intervention for enhancing sleep quality in adults. Moving forward, research should focus on conducting large-scale, long-term studies to determine the sustained effects of these interventions, as well as exploring demographic and psychological factors that might influence their efficacy. Additionally, the development of standardized, evidence-based protocols will be critical for optimizing the application of breathing exercises in clinical practice and daily routines. With continued research, breathing exercises have the potential to become a cornerstone of sleep quality improvement strategies, offering a holistic approach to better sleep and overall wellbeing.
